# Prevalence of Hyponatremia in Intensive Care Unit Patients With Brain Injury in Kashan Shahid-Beheshti Hospital in 2012

**DOI:** 10.5812/atr.9877

**Published:** 2013-08-01

**Authors:** Zahra Chitsazian, Batool Zamani, Maryam Mohagheghfar

**Affiliations:** 1 Department of Internal Medicine, Kashan University of Medical Sciences, Kashan, IR Iran; 2 Department of Internal Medicine, Kashan University of Medical Sciences, Kashan, IR Iran

**Keywords:** Hyponatremia, Brain Injury, Intensive Care Unit

## Abstract

**Background:**

Hyponatremia is a common disorder in patients with brain injury. It can result in acute and chronic complications providing this electrolytic disorder is not diagnosed and treated in due time.

**Objectives:**

The aim of this study was to evaluate the prevalence of hyponatremia in 95 brain injury patients hospitalized in the intensive care unit (ICU) in Kashan Shahid-Veheshti hospital.

**Patients and Methods:**

This trans-sectional study was conducted on brain injury patients (brain traumas, brain hemorrhage, meningitis and brain tumors) during their six-month stay in the ICU in Kashan Shahid-Beheshti hospital. Data were analyzed after excluding cases of pseudohyponatremia.

**Results:**

Ninety-five patients with brain injury (69.5% male and 30.5% female ( had a mean age of 42.85 ± 22.59 years, while the hyponatremic patients had a mean age of 48.37 ± 24.03 years. Prevalence and occurrence of hyponatremia were 31.6% and 9.29 ± 6.8 days, respectively. This study revealed no meaningful differences between age, sex, underlying disease and the prevalence of hyponatremia.

**Conclusions:**

Our study showed an elevated frequency of hyponatremia in patients with brain injuries in ICU which demands the effective approaches for an accurate and timely diagnosis of this electrolyte disorder.

## 1. Background

Sodium is the principal extracellular cation and main salt of osmolality (1). Sodium disorders are associated with considerable morbidity and mortality (2). Hyponatremia (plasma sodium concentration, less than 135 meq/L) is characterized by decreased osmolality. Antidiuretic hormone (ADH) and thirst are two main mechanisms for controlling body water regulation. Alternations in water and sodium balance affect the central nervous system (3). Disorder in water and sodium balance is a common finding in hospitalized patients and is especially very common among critically ill neurological patients due to the vital role of central nervous system in the regulation of water and sodium homeostasis (4). Arief reported a 7 - 60% prevalence of hyponatremia in a hospitalized group of patients and this was associated with a 1 - 15% increase in mortality (5). Acute hyponatremia causes more mortalities than chronic situations (6). Hyponatremia is mostly common among neurological patients than others hospitalized, and is especially characterized by sub-arachnoid hemorrhage (SAH), brain injuries due to head traumas, and meningitis (7). Sodium disorder is a potentially life-threatening situation and the rapid correction of hyponatremia is of great importance (8). The level of sodium concentration in hyponatremic patients is usually 120 - 130 mmol/L. Hyponatremia may contribute to neurological problems such as dizziness, lethargy, seizure, and finally coma (9). Early diagnosis and treatment can prevent complications such as worsening condition of the neurological patient, prolonged hospital stay, and rate of mortalities. The syndrome of inappropriate secretion of antidiuretic hormone (SIADH) is considered as one of the main causes of hyponatremia among adult neurological patients. Hyponatremia can also be due to the syndrome of cerebral salt wasting (CSW) characterized by polyuria and natriuresis because of intracerebral problems. It seems that higher atrial natriuretic peptide (ANP) and brain natriuretic peptide (BNP) levels are associated with increased hyponatremia and natriuresis in patients with acute brain injury especially following subarachnoid hemorrhage (SAH) (9). Administration of hypotonic fluids is among the main causes of iatrogenic hyponatremia in hospitalized cases (5, 10). Hyponatremia can also affect patients receiving normal saline or may arise due to preoperative ADH as a stress response. SIADH is associated with various causes. The major four groups of causes include neoplasia, non-malignant pulmonary diseases, drugs, and neurological diseases. The main neurological causes are meningitis, encephalitis, brain tumors, SAH, and brain traumatic injuries. CSWS has been mostly linked with SAH and brain traumatic injuries, tumors, tuberculosis, or carcinomatosis meningitis (9).

## 2. Objectives

Since many patients may likely experience acute and chronic complications due to a lack of accurate and timely diagnosis of sodium changes, we carried out this study to evaluate the prevalence of hyponatremia, its related causes, and its relationship with types of brain injury in patients with brain injuries admitted to the ICU of Shahid Beheshti hospital of Kashan in 2011.

## 3. Patients and Methods

In this trans-sectional study, patients with brain traumas, subarachnoid hemorrhage (SAH), intracranial hemorrhage (ICH), meningitis, and brain tumors, hospitalized from June 2011 to November of 2011 in ICU of Kashan Shahid-Beheshti hospital, were studied. Serum level of sodium was measured daily and serum levels less than 135 meq/L were diagnosed as hyponatremic and entered into the study. Demographic characteristics including age, sex, duration of ICU stay and day of hyponatremia manifestation, receiving or not receiving mannitol (to rule out hyponatremia), type of brain injury, levels of serum Creatinine and fasting glucose were gained from the patients’ files and recorded in the questionnaires. Complimentary tests, like blood lipid, triglyceride and cholesterol, thyroid hormones (TSH-T4), and urine samples for sodium and special gravity were done for confirming hyponatremia and investigating for its cause. Presence or absence of peripheral edema and heart failure were determined through previous examination and history taking while urine output of each patient was recorded within the past 24 hrs. Data were analyzed using SPSS software,T-test, Chi square , Leven and Fisher exact tests.

## 4. Results

The current study attempted to focus on the prevalence of hyponatremia in 95 brain injury patients admitted to ICU of Kashan Shahid Beheshti hospital in a six-month period since June 2011, of which 29 (30.5%) were female and 66 (69.5%) were male. Mean age in the hyponatremic patients was 48.37 ± 24.03 years, while this was 40.31 ± 21.6 years for the rest. Mean time for manifestation of hyponatremia was 9.29 ± 6.8 days. Thirty of the patients had hyponatremia with a prevalence of 31.6%; this consisted of 30.3% for males and 34.5% for the females. Based on the statistical analysis, hyponatremic prevalence showed no significant correlation with age, sex, and type of brain injury (brain hemorrhage, contusion, meningitis, and brain tumors) (Table 1 and Table 2). 

**Table 1.** Distribution Prevalence of Hyponatremia in the Brain Injury Patients by Sex and Underlying Disease

Variables	Affected, No. (%)	Not Affected, No. (%)	Total, No. (%)	P value
**Gender**				
**Male**	20 (30.3)	46 (69.7)	66 (100)	0.68
**Female**	10 (34.5)	19 (65.5)	29 (100)	
**Underlying **				
**With**	6 (33.3)	12 (66.7)	18 (100)	0.85
**Without**	24 (31.2)	53 (68.8)	77 (100)	

**Table 2.** Distribution Prevalence of Hyponatremia in Brain Injury Patients by Type

Brain Injury	Affected, No. (%)	Not Affected, No. (%)	Total, No. (%)	P value
**Brain Tumor**				
**With**	3 (18.8)	13 (81.3)	16 (100)	0.22
**Without**	27 (34.2)	52 (68.8)	79 (100)	
**Hemorrhage**				
**With**	22 (37.3)	37 (62.7)	59 (100)	0.12
**Without**	8 (22.2)	28 (77.8)	36 (100)	
**Contusion and Trauma**				
**With**	13 (40.6)	19 (59.4)	32 (100)	0.17
**Without**	17 (27)	46 (73)	63 (100)	
**Meningitis and Encephalitis**				
**With**	1 (100)	0 (0)	1	0.31
**Without**	29 (30.9)	65 (69.1)	94 (100)	

## 5. Discussion

This study was conducted to evaluate the prevalence of hyponatremia in 95 patients with various types of brain injuries (including brain tumors, hemorrhage, contusion, and meningitis) admitted to ICU of Shahid Beheshti hospital during a six-month period. Hyponatremia in the study showed a high prevalence of 31.6%. According to the results, early days in the second week of hospitalization are the most probable time for development of hyponatremia in patients. In a study on 26 brain injury patients hospitalized during 2004 - 2005 in the intensive care unit in Brazil, Karina et al. observed a 34.6% prevalence of hyponatremia in the patients, which was quite consistent with that of ours (2). Sherlock et al. directed a study on 1695 patients undergoing brain surgery in Beaumont hospital, Ireland, in 2009 in which 11% of the patients with brain tumor, hemorrhage, and brain tumor had sodium excretion of less than 130. This rate of prevalence was somewhat lower than that obtained by us which may be due to the different definitions for hyponatremia in both studies. Our study considers a patient with sodium less than 135 as hyponatremic, while Sherlock and colleagues states that an excretion of sodium less than 130 is enough for a patient to be considered as hyponatremic which justifies the higher prevalence of hyponatremia in our study. Another cause for this high prevalence in our study can be because of the severity of brain injury in our patients which were all referred to the ICU, whereas Sherlock covered only those brain injury patients received by the hospital (11). In another study, carried out in 2004 by Nobohiro et al. to evaluate the prevalence of hyponatremia and response to sodium supplement, 16.8% of the 298 patients with traumatic brain injuries were found to be hyponatremic which was less than that reported by us. The reason may be due to the different definitions available for hyponatremia, focusing only on traumatic brain injury patients in the aforementioned study and not covering other brain injury patients, or severity of the brain injury (12). Born et al. reported that out of 109 patients with severe trauma, 36 (33%) were hyponatremic which was consistent with the prevalence found by our study (31.6%) (13). In a study that Upadhyay and colleagues performed on some hospitalized patients, it was revealed that in 28.2% of the patients at least one patient had hyponatremia. This rate was higher for those who stayed in the ICU (18 - 30%), which was in line with our study (14). In another study conducted by Saboori et al. during 2002 - 2003 on 100 patients admitted to the neurosurgical centers of Kashani and Alzahra hospitals in Isfahan, a 42% prevalence of hyponatremia was observed (15). The elevated prevalence reported in his study compared to that of ours may be the result of difference in sample sizes and severity of the brain injury. Cerda et al. in a research done in 2010, titled as “Prevalence and Causes of Hyponatremia in the Neurologic Patients” showed that from the 130 patients under study, 14.6% were hyponatremic (16). In the current study, mean time for manifestation of hyponatremia was 9.29 (SD = 6.8) days proving that hyponatremia appears mostly within the second week of hospital stay. The study carried out by Nobohiro et al. on 298 patients with brain traumatic injuries during 2003 - 2004 revealed that hyponatremia had developed three to eight days following the injury (12). In the study done by Karina et al on 26 brain injury patients admitted to the ICU of a Brazilian hospital, it was detected that hyponatremia was present mostly during days 2 - 10 of hospital stay among which the sixth and seventh days were the most probable days for hyponatremia. The most probable times for manifestation, in their study was late in the first week and early in the second week of hospital stay. Our study confirms early in the second week as the most prevalent time for hyponatremia manifestation (2). Studying 1698 patients with brain injury in a hospital in Ireland in 2009, Sherlock et al. concluded that the mean time for manifestation of hyponatremia was 6.7 days following the injury. This study considers the time after the first week as the most prevalent time for hyponatremia development which is consistent with our study (11). Mean age for the hyponatremic patients in our study was 48.37 years. No meaningful correlation between mean age and hyponatremia in the patients was detected in the current study. Upadhyay et al. in 2006 reported a mean age of 47 ± 10 years for the hyponatremic patients which is consistent with our study (14). Sherlock et al. in a study on 1698 patients with brain injury, in 2009, recorded a 41.7-year mean age for the hyponatremic patients. Their study showed no correlation between age and hyponatremia (11). In the study carried out by Karina et al. on patients admitted to the ICU of a hospital in Brazil, the cases had at most between 18 to 54 years of age (2).

Prevalence of hyponatremia in the cases with brain tumors, brain hemorrhage, and brain tumours was evaluated as 18.8%, 37.3%, and 40.6%, respectively in our study. Only one patient with meningitis was detected which had hyponatremia. In the study by Sherlock et al. in Beaumont hospital in 2009, prevalence of hyponatremia in the patients with traumatic brain injury, Intracerebral neoplasma, and subarachnoid hemorrhage was 44.4 - 9.6%, 15.8 1 -56.3%, and 19.6 - 62.3%, respectively (11). Mulliy et al. in a study titled “Hyponatremic Emergencies” found that hyponatremia is present in 7 - 32% of patients with meningitis (15). A study titled as “Prevalence and Pathophysiology of Hyponatremia” performed by Sherlock et al. in 2006 confirmed that 56% of patients with SAH were hyponatremic (17). It seems that the differences in the prevalence rate of hyponatremia among patients with different brain injuries might be due to the kind of serum and drugs received, severity of the brain injury-induced hyponatremia, different definitions for hyponatremia, or difference in the day for measuring sodium serum level than the primary brain injury. This study detected no significant statistical difference between prevalence of hyponatremia and age, sex, underlying disease, and type of brain injury. Our study demonstrated an elevated prevalence for hyponatremia in brain injury patients including types of brain tumors, brain hemorrhage, meningitis, and contusion. The most prevalent time for hyponatremia manifestation was during the second week of hospital stay. Since hyponatremia can result in chronic complications such as cerebral edema, reduced cerebral perfusion, worse prognosis, and prolonged hospital stay in these patients, thus, continuous follow-up, early diagnosis, and more accurate actions are recommended for treatment and prevention of hyponatremia for ICU patients.
